# Development of Olaparib-Resistance Prostate Cancer Cell Lines to Identify Mechanisms Associated with Acquired Resistance

**DOI:** 10.3390/cancers14163877

**Published:** 2022-08-11

**Authors:** Maxime Cahuzac, Benjamin Péant, Anne-Marie Mes-Masson, Fred Saad

**Affiliations:** 1 Centre de Recherche du Centre Hospitalier de l’Université de Montréal (CRCHUM), Montreal, QC H2X 0A9, Canada; 2 Institut du Cancer de Montréal, Montreal, QC H2X0A9, Canada; 3 Department of Medicine, Université de Montréal, Montreal, QC H3T 1J4, Canada; 4 Department of Surgery, Université de Montréal, Montreal, QC H3T 1J4, Canada

**Keywords:** PARP, resistance, prostate cancer, DNA repair, autophagy

## Abstract

**Simple Summary:**

PARP inhibitors (PARPi; olaparib) are presently in clinical trials for advanced prostate cancer (PC). Resistance mechanisms are not fully understood in PC compared to ovarian and breast cancers. Our study aimed to identify new molecular mechanisms that affect acquired olaparib-resistance. We developed new resistant PC cell line models derived from original PC cell lines. We identified that DNA repair, autophagy, and the Rho-associated coiled-coil containing protein kinase 2 (ROCK2) could be potential targets to reverse the acquired olaparib-resistance.

**Abstract:**

Background: Poly (ADP-ribose) polymerase inhibitors (PARPi) were initially deployed to target breast and ovarian tumors with mutations in DNA damage response genes. Recently, PARPi have been shown to be beneficial in the treatment of prostate cancer (PC) patients having exhausted conventional therapeutics. Despite demonstrating promising response rates, all patients treated with PARPi eventually develop resistance. However, PARPi resistance in PC is not well understood, and further studies are required to understand PARPi resistance in PC to propose strategies to circumvent resistance. Methods: Starting from well-established olaparib-sensitive PC cell lines (LNCaP, C4-2B and DU145), we derived olaparib-resistant (OR) PC cell lines and performed a microarray analysis. Results: The olaparib IC50 values of OR cell lines increased significantly as compared to the parental cell lines. Gene expression analyses revealed that different pathways, including DNA repair, cell cycle regulation and autophagy, were affected by acquired resistance. A total of 195 and 87 genes were significantly upregulated and downregulated, respectively, in all three OR cell lines compared to their parental counterparts. Among these genes, we selected BRCC3, ROCK2 and ATG2B for validation. We showed that ROCK2 expression, basal autophagy and homologous recombination (HR) efficiency were increased in all OR cell lines. Conclusions: Our study provides a new in vitro model to study PARPi resistance in PC and suggests new possible targets to reverse resistance and prolong the benefits of PARPi treatment.

## 1. Introduction

Advances in next-generation hormonal therapy (NHT), such as enzalutamide and abiraterone, or chemotherapy with docetaxel, have helped to improve overall survival in prostate cancer (PC). However, resistance to these therapies is common and associated with an increased risk of aggressive metastatic disease for which effective treatments are limited. Poly (ADP-ribose) polymerase (PARP) inhibitors (PARPi) are a novel class of anticancer therapeutics that have become part of standard treatment for breast and ovarian cancers carrying BRCA mutations and represent a therapeutic option for treatment-refractory PC. PARPi treatment induces synthetic lethality in tumors carrying mutations in DNA damage response (DDR) genes such as BRCA1, BRCA2 or ATM, which are found to be somatically mutated in approximately 30% of metastatic PC patients [1,2]. Ongoing clinical trials using PARPi in PC as monotherapy or in combination with next-generation hormonal therapy have shown potential benefits for patients [3,4]. The PROfound study included men with HR mutations that had progressed on at least one line of NHT. Patients were randomized to either the PARPi olaparib or the physician’s choice of an NHT [3]. The study reported a significant improvement in radiographic progression-free survival (rPFS) and overall survival even though cross-over was allowed at the time of progression. This led to the first international approval for the use of a PARPi in PC. The PROpel clinical trial included patients undergoing treatment for first-line metastatic castration-resistant PC [4]. The study compared the combination of olaparib with abiraterone versus abiraterone alone in patients and demonstrated a significant improvement in rPFS in the combination arm (24.8 months vs. 16.6 months, respectively) [5]. Despite these promising outcomes, the development of PARPi resistance needs to be better understood to further improve the outcomes of patients treated with PARPi.

PARPi target PARP1/2 enzymes, which are essential for DNA repair mechanisms, especially base excision repair (BER). The inhibition of BER results in the transformation of single-strand break to double-strand break, which is often not resolved in homologous recombination (HR)-deficient tumors leading to cell death. Innate resistance to PARPi is mainly associated with the absence of mutations that impair HR [6,7]. Other mechanisms, such as autophagy, may also contribute to de novo resistance to PARPi [8,9,10]. In breast and ovarian cancer, acquired resistance can occur via several mechanisms, including the restoration of HR, the stabilization of the replication fork, or alterations in drug target and transport [11]. It has been observed that prolonged PARPi treatment of breast and ovarian cancer results in the selection of cells that reverse mutations in BRCA1 and BRCA2 genes to restore the functionality of HR and reduce the cytotoxic effects of PARPi [12,13]. Some mutations were also observed in the catalytic site (K199 and S120) of PARP1 to limit PARPi-binding and PARP1-trapping on DNA [14]. Studies also suggest that cancer cells will decrease the activity of the EZH2 enzyme and PTIP binding to limit the collapse of the replication fork and chromosomal instability [15]. In PC, resistance mechanisms are not well understood or studied.

Here, we developed three olaparib-resistant (OR) PC cell lines by exposing LNCaP, C4-2B and DU145 PC cells to increasing concentrations of olaparib over a period of six months. PC-OR cell lines harbored a higher resistant profile compared to the wild type (WT) PC cells without affecting their doubling time or morphology. Microarray experiments were performed and allowed a transcriptome analysis between olaparib-sensitive and -resistance cell lines. In addition to identifying pathways and genes potentially implicated in acquired resistance in PC, this study also provides useful models for future studies on acquired olaparib-resistance in PC.

## 2. Materials and Methods

### 2.1. Cell Culture and Generation of OR Cell Lines

Human PC cell lines, LNCaP (RRID:CVCL_0395) and DU145 (RRID:CVCL_0105), were purchased from the American Type Culture Collection (Manassas, VA, USA; ATCC CRL-174 and ATCC HTB- 81, respectively). The LNCaP C4-2B, denoted C4-2B in the study, (RRID:CVCL_4784) cell line was kindly gifted by Dr. Martin Gleave (Vancouver Prostate Centre, BC, Canada). All cell lines were verified by short tandem repeat (STR). Cell lines were cultured in RPMI 1640 medium (Wisent Inc., St-Bruno, QC, Canada; 350-000-EL) supplemented with 10% fetal bovine serum (FBS; Wisent Inc., St-Bruno, QC, Canada; 098–150), 0.5 µg/mL amphotericin B (Wisent Inc., St-Bruno, QC, Canada; 450–105-QL), and 50 µg/mL gentamicin (Life Technologies Inc., Carlsbad, CA, USA; 15710064). All cells were grown in 5% CO2 at 37 °C. OR cells derived from LNCaP, C4-2B and DU145 were obtained by culturing parental cell lines in increasing concentrations (0.5 to 70 µM) of olaparib (Selleckchem, Conshohocken, PA, USA; AZD2281) for 6 months. Doubling time and cell morphology were determined using the IncuCyte^TM^ Live-Cell Imaging System (Essen BioScience, Inc., Ann Arbor, MI, USA).

### 2.2. Clonogenic Assay

LNCaP WT/-OR were seeded at 1000 cells/well while C4-2B WT/-OR and DU145 WT/-OR were seeded at 500 cells/well in 6-well plates. The next day medium was removed and replaced with RPMI complete medium containing olaparib (from 0.00125 to 40 µM). After 7 days of treatment, cells were fixed with methanol and stained with a solution of 50% (*v*/*v*) methanol and 0.5% (*m*/*v*) blue methylene (Sigma-Aldrich Inc., Saint-Louis, MO, USA). Colonies were counted under a stereomicroscope and reported as a percentage of the control. IC50 values were determined using Graph Pad Prism 9 software (GraphPad Software Inc., San Diego, CA, USA). Each experiment was performed in duplicate and repeated three times.

### 2.3. RNA Preparation and Microarray Analyses

RNA from WT and OR cell lines (LNCaP, C4-2B and DU145) was extracted as described previously [16]. Gene expression microarray experiments were performed at the McGill Genome Centre (mcgillgenomecentre.ca) using human-Clariom S arrays (Affymetrix^®^, 902927). Data were analyzed using the Transcriptome Analysis Console (Affymetrix^®^). To determine enriched pathways, GSEA analysis was performed using the GSEA 4.2.1 software (Broad Institute, Harvard, UK). The Gene Ontology Biological Process (GOBP) was used as the gene set database, and signal-to-noise ratio was selected for ranking gene metric parameters. Bubble charts were created using the R software and ggplot2 extension [17]. Volcano plots were created using the web app VolcaNoseR [18]. Downregulated genes with a fold change(log2) ≤ −0.6 and upregulated genes with a fold change(log2) ≥ +0.6 with a significant (log-10) q-value ≥ 1.3 were used as cut-offs for the study. Genes included in these thresholds were visualized by creating a heatmap using Heatmapper [19].

### 2.4. Real-Time q-PCR

Total RNA was extracted from PC WT and OR cell lines using the RNeasy kit (Qiagen Inc., Hilden, Germany, 74106). A total of 1 µg of RNA was used for the reverse transcription using the QuantiTect Reverse Transcription Kit (Qiagen Inc., Hilden, Germany, 205313). A total of 1 µL of diluted (1:10) reverse-transcribed products was used for Q-PCR using sequence specific primers (400 nM) and the SYBR Select Master Mix (Applied Biosystems^®^, Life Technologies Inc., Carslbad, CA, USA, 4472908). Sequence primers for target genes, *ATG2B*, *BRCC3, ROCK2* and *Actin* are available in Table 1. Q-PCR were performed using Applied 63 BioSystems Step One Plus system (UDG activation at 50 °C/2 min, followed by the AmpliTaq activation plus denaturation cycle at 95 °C/2 min, followed by 40 cycles at 95 °C/15 s, 60 °C/1 min, and 72 °C/30 s. Gene expression values were normalized to *ß-actin* gene expression.

### 2.5. Protein Preparation and Western Blot Aalysis

Proteins were extracted from cell lines using mammalian protein extraction reagent (MPER; 50 nM Tris-HCl, 200 mM NaCl, 0.25% Triton 100X, and 10% glycerol) containing a protease and phosphatase inhibitor cocktail (ThermoFisher, Waltham, MA, USA; PIA32961). Protein concentration was determined by Bradford assay (Bio-Rad Laboratories, Hercules, CA, USA; 500–0006). Twenty micrograms of protein were separated in precast 4–15% gradient Tris-glycine SDS-polyacrylamide gels (Mini-PROTEAN^®^ TGXTM Precast Gels, Bio-Rad, Hercules, CA, USA; 456–1086) and transferred in Trans-Blot Turbo Mini 0.2 μm nitrocellulose membranes (Bio-Rad, Hercules, CA, USA; 170–4159). Membranes were blocked with 5% milk in PBS-Tween for 1 h and incubated overnight at 4 °C with primary antibody in BSA 1%. Membranes were next incubated with secondary antibody for 1 h, and signal was detected using the ChemiDoc MP Imaging System (Bio-Rad, Hercules, CA, USA). ß-Actin was used as a loading protein control and each experiment was repeated three times. Image J [20] was used to quantify protein levels in Western blots.

### 2.6. Antibodies

ß-Actin (AC14) (abcam, Cambridge, UK; AB6276, 1:20,000 dilution), PARP1 (Proteintech, Rosemont, IL, USA; 66250, 1:650 dilution), PAR/pADR (R&D systems, Minneapolis, MN, USA; 4335-MC-100, 1:1000 dilution), goat anti-mouse (Millipore, Burlington, MA, USA; AP124P, 1:4000 dilution), goat anti-rabbit (Millipore, Burlington, MA, USA; AP156P, 1:10,000 dilution) and rabbit anti-goat (Millipore, AP106P, 1/4000 dilution).

### 2.7. Measurement of Autophagic Flux

Autophagic flux was measured as described previously [8]. Briefly, 15,000 to 25,000 cells were seeded in 24-well plates with coverslips and transfected with the Premo Autophagy Tandem Sensor RFP-GFP-LC3B BacMam 2.0 Expression vector (Thermo Fisher, Waltham, MA, USA; P36239) according to manufacturer’s protocol. The 24 h post-transfection cells were fixed with formalin for 15 min. Prolong Gold^®^ anti-fade reagent with DAPI (Life Technologies Inc., Carlsbad, CA, USA; 14209 S) was used for mounted coverslips onto slides.

### 2.8. Analysis of HR and NHEJ Activity

HR and NHEJ activities were measured using the reporter plasmids pcDNA-GFP HR and pCDNA-GFP NHEJ, a gift from Dr. Jean-Yves Masson (Université de Laval, QC, Canada), as described previously [8]. Briefly, 200,000 cells were seeded in 6-well plates and transfected 24 h using Lipofectamine 3000 (Thermo Fisher Scientific, Waltham, MA, USA;) with the reporter plasmid and pCMV-I-SceI vector containing an mCherry-tag. The 48 h post-transfection cells were processed, and the ratio of GFP-positive (Q2) and mCherry-positive cells (Q3) was determined by Fortessa flow cytometer (BD Biosciences).

### 2.9. Statistics

Statistical analyses were performed using Graph Pad Prism 9 (GraphPad Software Inc., San Diego, CA, USA) using the two-tail Student *t*-test, which was justified appropriately for every experimental design. The data were normally distributed, and the variance between groups that were compared was similar. A *p* value of less than 0.05 was considered statistically significant. FDR q-value was automatically calculated during the GSEA analysis.

## 3. Results

### 3.1. Generation of PC Cell Lines with Acquired Resistance to PARPi Olaparib

**Figure 1:**
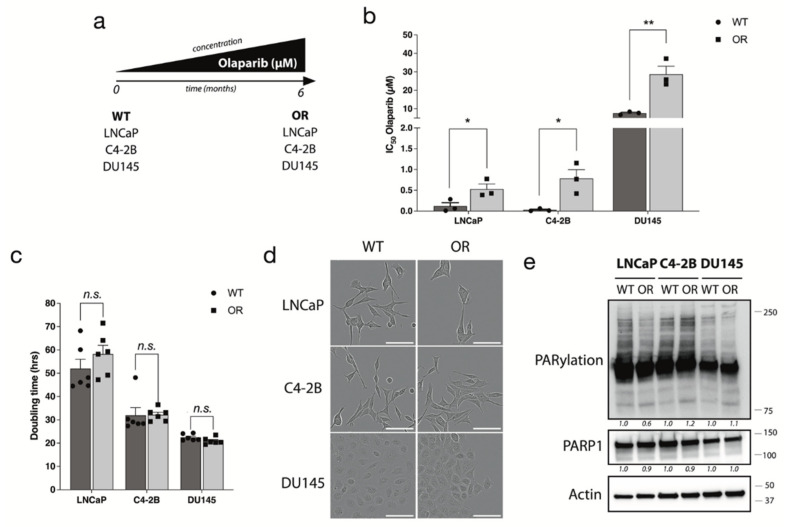
Development of PC cell lines resistant to olaparib (OR) from parental cell lines. (**a**) Schematic representation of PC-OR cell lines development. (**b**) Olaparib IC50 of PC WT and OR cell lines. (**c**) Doubling time of WT and OR cell lines. (**d**) Representative images of WT and OR cell morphology after 4 days of culture. (**e**) PARP1 expression and PARylation activity measured by Western blot on total cell extracts from WT and OR PC cell lines. Actin was used as loading control. For all data, the mean ± SEM of three independent experiments is shown. Data were analyzed using the two-tail Student *t*-test. *n.s.* = non-significant. * *p* <  0.05, ** *p*  <  0.01.

To characterize mechanisms that contribute to acquired PARPi resistance, we cultured three PC cell lines, LNCaP, C4-2B and DU145, in medium containing increasing olaparib concentrations for six months (Figure 1a). Compared to their respective parental cell line, the olaparib IC50 values significantly increased for all three OR cell lines: 4.41-fold change for LNCaP-OR (*p* = 0.05), 28.9-fold change for C4-2B-OR (*p* = 0.024), and 3.78-fold change for DU145-OR (*p* = 0.0080) (Figure 1b and Table 2). Doubling time and cell morphology were not changed by the acquisition of olaparib resistance (Figure 1c,d). Since PARPi inhibits PARylation by PARP1, the expression of PARP1 and its PARylated forms were evaluated by Western blot (Figure 1e and Figure S3a). OR cell lines showed a slight decrease in PARylation, although PARP1 expression was not affected compared to WT.

### 3.2. Identification of Pathways Involved in Olaparib Resistance by Microarray Analysis

**Figure 2:**
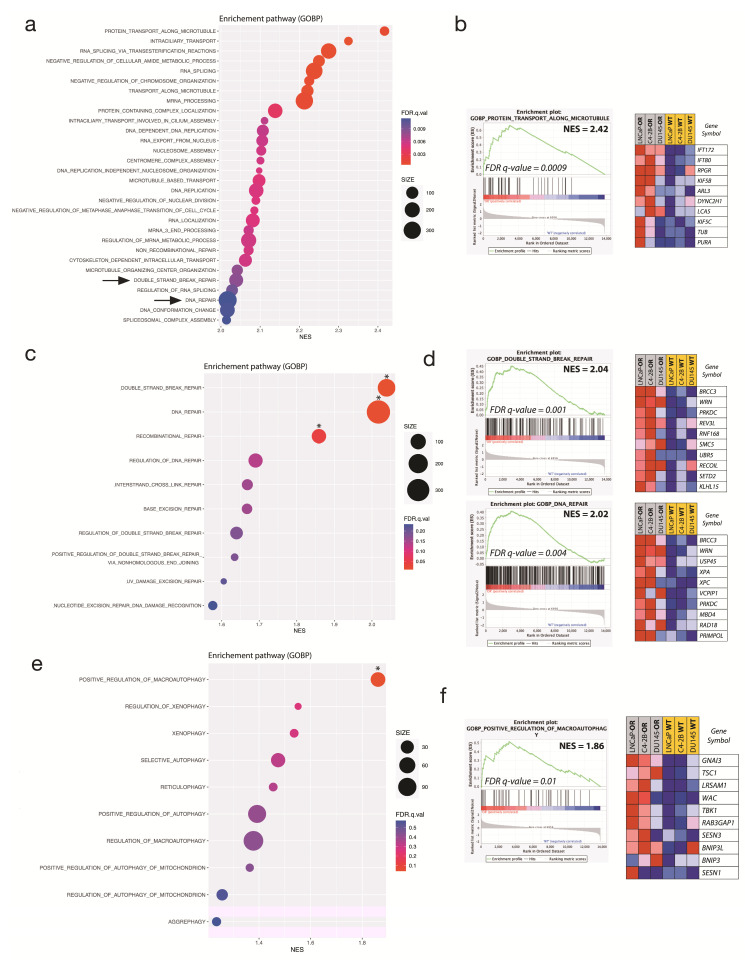
Enrichment pathways in PC-OR cell lines. (**a**) Bubble chart regrouping the 30 most significantly enriched pathways determined by GSEA analysis. (**b**) Enrichment plot and top 10 enriched genes in the “protein transport along microtubules”. (**c**) Bubble chart derived from the initial analysis (**a**) and regrouping the top 10 enrichment pathways linked to DNA repair mechanisms. (**d**) Enrichment plot and top 10 enriched genes of “double strand break repair” and “DNA repair” pathways. (**e**) Bubble chart derived from the initial analysis in (**a**) and regrouping the top 10 enrichment pathways linked to autophagy mechanisms. (**f**) Enrichment plot and top 10 enriched genes of “positive regulation of macroautophagy” pathway. * *p* < 0.05.

**Figure 3:**
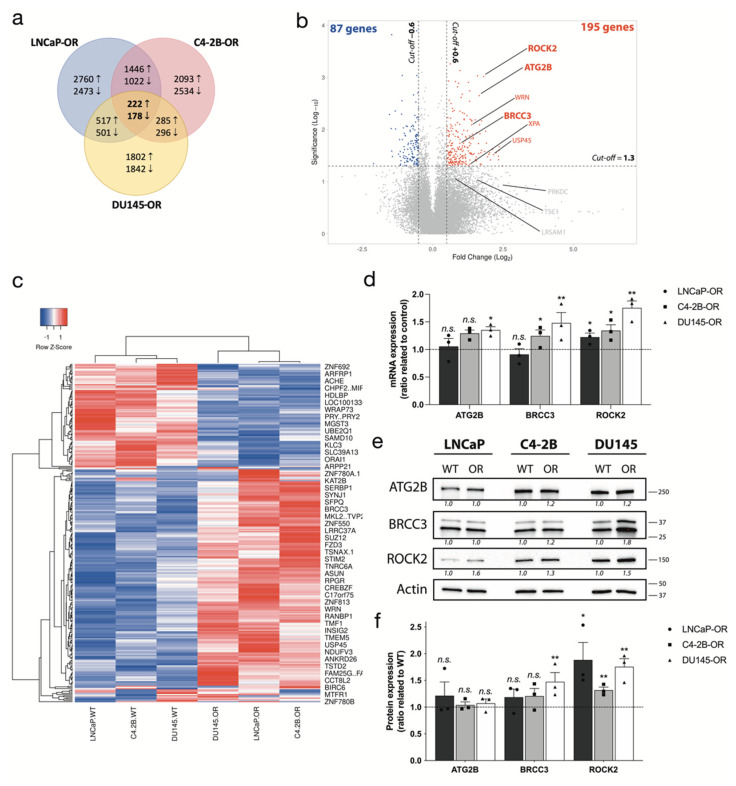
Determination of the most upregulated and downregulated genes in all three PC-OR cell lines. (**a**) Venn diagram representing all genes that were upregulated (222) and downregulated (178) in all OR cell lines compared to all WT cell lines. (**b**) Volcano plot with fold change(log2) ≤ −0.6 and with a fold change(log2) ≥ +0.6 as thresholds for gene expression, and (log−10) *q*-value ≥ 1.3 as the threshold for statistically significant variation of expression. A total of 195 upregulated (red) and 87 downregulated (blue) genes were identified using these thresholds. (**c**) Heatmap showing all significantly upregulated genes in red and all significantly downregulated genes in blue. (**d**) Validation of ATG2B, BRCC3 and ROCK2 RNA expression by RT-qPCR. (**e**) Validation of ATG2B, BRCC3 and ROCK2 protein expression by Western blot. Actin was used as loading control. (**f**) Quantitation of (**e**). For all data, the mean ± SEM of three independent experiments is shown. Data were analyzed using the two-tail Student *t*-test. *n.s.* = non-significant. * *p* < 0.05 and ** *p* < 0.01.

To identify genes and pathways that were associated with acquired olaparib resistance, we performed microarray analysis on our three WT and OR PC cell lines using Illumina Sentrix (Figure 2 and Figure 3). Gene set enrichment analysis (GSEA) showed that OR cell lines were significantly enriched in pathways involved in “protein transport along microtubule” (false discovery rate (*FDR*) *q*-value = 0.0009), RNA splicing (*FDR q*-value = 0.001) and DNA repair (*FDR q*-value = 0.01), all of which had a normalized enrichment score (NES) above 2.0 (Figure 2a). Heatmap of enriched genes for “protein transport along microtubule” showed that genes for intraflagellar transport 172 and 80 (*IFT172/80*) were upregulated in OR cell lines (Figure 2b). Because regulation of DNA repair is one mechanism for PARPi resistance, we selected the top 10 DNA repair mechanisms (Figure 2b,c). “double-strand break repair” (*FDR q*-value = 0.0083, NES = 2.04), “DNA repair” and “recombinational repair” (*FDR q*-value = 0.040, NES = 1.86) were associated with a higher NES. “Non-homologous end-joining” (NHEJ) and “single strand DNA break repair” pathways such as base or nucleotide excision repair (BER/NER) were not affected by the acquisition of olaparib resistance. GSEA analysis also revealed that BRCA1/BRCA2-Containing Complex Subunit 3 (*BRCC3*) and Werner syndrome helicase (*WRN*) were upregulated in all three OR cell lines compared to WT (Figure 2d).

We also interrogated the top 10 pathways involved in autophagy, as this process has also been reported to induce PARPi resistance (Figure 2e). The GSEA gene set for “positive regulation of macroautophagy” was the only pathway that was significantly impacted (*FDR q*-value = 0.040), with an NES = 1.86. This pathway includes genes encoding for protein that increase levels of autophagy such as beclin-1 (*BECN1*), *ATG2B*, or components of the mTOR complex. Among these genes, G protein subunit alpha i3 (*GNAI3*) and TSC complex subunit 1 (*TSC1*) were upregulated in OR PC cell lines (Figure 2f).

This first overview analysis showed that protein transport along microtubule, autophagy, and DNA repair mechanisms, especially recombinational repair, were significantly altered in olaparib-resistant PC cell lines.

### 3.3. Determination of Common Genes between OR Cell Lines

To further analyze the genes that were associated with olaparib resistance, the list of differentially expressed genes of the three OR cell lines was compared and revealed that all three shared 222 genes that were upregulated and 178 genes that were downregulated (Figure 3a). These genes were entered in a volcano plot to select only genes that had a *p*-value log(−10) ≥ 1.3 and a fold-change (log2) ≤ −0.6 and ≥ +0.6 (Figure 3b). A total of 195 upregulated and 87 downregulated genes were obtained by applying these cut-offs (Figure 3c and Supplementary Figure S1). The top 20 upregulated and downregulated genes were classified by their significance (Table 3 and Table 4). Fold enrichment analysis showed that genes regulating cell cycle progression, such as Rho-associated coiled-coil containing protein kinase 2 (*ROCK2*) or Cyclin-dependent kinase 12 (*CDK12*), and genes regulating RNA processing, such as mago homolog exon junction complex subunit (*MAGOH*) or SR-related CTD associated factor 11 (*SCAF1*) were the most represented among the 195 upregulated genes (Supplementary Figure S2). *ATG2B* from the autophagy pathway was also significantly upregulated by a 1.65-fold change (log2) (*p*-value log(−10) = 2.65). Other genes, such as *GNAI3* or *TSC1*, found in the GSEA gene set of “positive regulation of macroautophagy” were not significantly affected. Genes involved in DNA repair mechanisms, such as *RAD54B* (*p*-value log(−10) = 2.54), were also significantly upregulated in OR cell lines. We classified genes for the “DNA repair” pathway obtained from our GSEA analysis based on the order of enrichment (Table 5 and Figure 2c). Not all genes were impacted by the acquisition of the OR phenotype. Only *BRCC3* (*p*-value log(−10) = 1.87), *WRN* (*p*-value log(−10) = 2.09), *USP45* (*p*-value log(−10) = 1.52) and the xeroderma pigmentosum complementation group A (*XPA*; *p*-value log(−10) = 1.32) were significantly upregulated. No pathways seemed significantly enriched in the 87 downregulated genes. Genes that were significantly downregulated included GRAM domain containing 4 (*GRAMD4*; *p*-value log(−10) = 3.82) followed by H2A clustered histone 17 (*HIST1H2AM*; *p*-value log(−10) = 3.03) and Keratin associated protein 21–1 (*KRTAP21-1*; *p*-value log(−10) = 2.66). Overall, DNA repair mechanisms, autophagy but also apoptosis, cell cycle and mRNA processing may be involved in the acquisition of olaparib resistance in PC.

### 3.4. Validation of Selected Genes ATG2B, BRCC3 and ROCK2

We next decided to select three genes from our previous analyses depending on the literature and their role in autophagy, DNA repair and cell cycle regulation (*ATG2B*, *BRCC3* and *ROCK2,* respectively) [21,22,23]. ATG2B RNA expression was significantly upregulated in DU145-OR only (1.4-fold change, *p* = 0.04), unlike protein expression, where no significant variation was observed (Figure 3d–f and Figure S3b,c). BRCC3 was also significantly increased in C4-2B-OR (1.3-fold change, *p* = 0.05) and DU145-OR (1.5-fold change, *p* = 0.003) for RNA and only in DU145-OR (1.5- fold change, *p* = 0.02) for protein. Interestingly, RNA and protein expression of ROCK2 were upregulated in all PC OR lines (protein level LNCaP-OR 1.8-fold change, *p* = 0.004; C4-2B-OR 1.3-fold change, *p* = 0.002; DU145-OR 1.7-fold change, *p* = 0.002).

### 3.5. PC OR Cell Lines Increase Autophagy and Homologous Recombination

**Figure 4:**
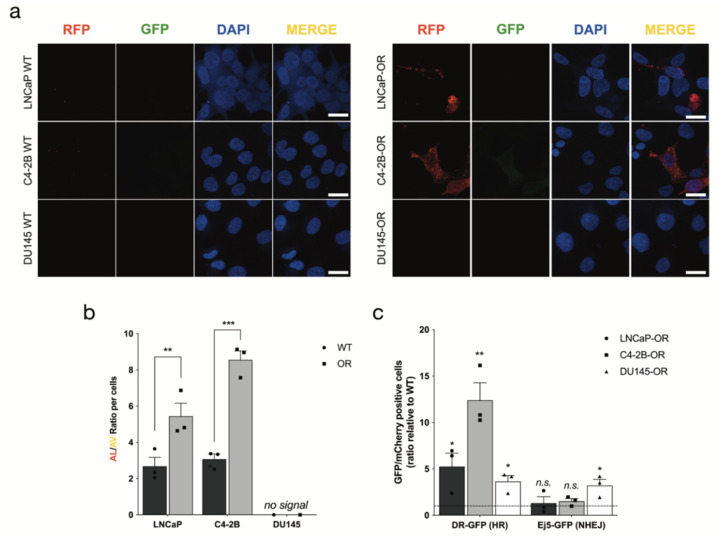
Autophagy and HR efficiency are upregulated in PC-OR cell lines. (**a**) Representative images of autophagy flux with the tandem RFP-GFP LC3B sensor in WT and OR PC cell lines, captured by confocal microscopy. (**b**) Quantification of (**a**) using ImageJ software to calculate the ratio between autolysosomes (AL; red puncta) and autophagosomes (AV; yellow puncta) relative to control. (**c**) Ratio of HR (DR-GFP) and NHEJ (Ej5-GFP) efficiency by flow cytometry compared to respective PC WT. For all data, the mean ± SEM of three independent experiments is shown. Data were analyzed using the two-tail Student *t*-test. *n.s.* = nonsignificant. * *p* < 0.05, ** *p* < 0.01 and *** *p* < 0.001.

To determine functional changes associated with PC OR, we measured autophagy and HR/NHEJ efficiency in PC-WT/-OR cell lines. We used the Premo^TM^ Autophagy Tandem Sensor construct containing the LC3-II protein tagged with red and green fluorescence protein (RFP/GFP) to measure the basal level of autophagy in our cell lines (Figure 4a,b). This method measures the autophagic flux (ratio of number of autolysosomes (AL) to autophagosome (AV)) by the loss of GFP signal induced by the fusion of lysosome and AV to form the AL, the last step of the autophagy mechanism. Interestingly, the basal level of autophagy was significantly increased in LNCaP-OR (2-fold change, *p* = 0.033) and C4-2B-OR (2.8-fold change, *p* = 0.0007) compared to respective PC WT cell lines (Figure 4b). No signal was observed in DU145 due to a lack of ATG5 expression and LC3-II formation [24]. HR (DR-GFP) and NHEJ (Ej5-GFP) efficiency were measured using the plasmid-based DNA repair GFP reporter assay described previously [8] (Figure 4c). All three PC-OR cell lines had an increase in HR efficiency compared to PC WT (LNCaP-OR 5.2-fold change, *p* = 0.04, C4-2B-OR 12.4-fold change, *p* = 0.004 and DU145-OR 3.6-fold change, *p* = 0.03). NHEJ was also increased but in DU145-OR only (3.2-fold change, *p* = 0.03). Therefore, PC-OR cell lines had a higher basal level of autophagy and HR efficiency compared to their WT counterparts.

## 4. Discussion

Mechanisms of PARPi antitumor activity and resistance are well described for breast and ovarian cancers. These inhibitors are also promising therapeutic agents to prolong life and ameliorate the response to commonly used therapies for PC patients. However, PARPi resistance is also a potential and expected risk that is not well understood in PC. In this study, we developed olaparib-resistant PC cell models derived from PC cell lines LNCaP, C4-2B and DU145 to identify genes and mechanisms involved in acquired resistance. Our analysis showed that several pathways, such as DNA repair or autophagy, may regulate olaparib resistance.

Our GSEA overview analysis showed that “double strand break repair” and “recombinational repair” were significantly enriched with an NES over 2.0. Genes from these pathways, such as *BRCC3*, *WRN*, *USP45* and *XPA*, were also found in our volcano plot analyses. BRCC3 and WRN are known to play a role in HR, USP45 and XPA in NER. Interestingly, these genes are linked to resistance to radio- and chemotherapies for cancer. Studies have shown that their expression is increased after treatment, inducing an increase in DNA repair and resulting in resistance to these treatments [25,26,27]. These results support the idea that PARPi-resistant cancer cells have altered DNA repair pathways to overcome the cytotoxic effect of olaparib. Our results revealed that PC-OR cell lines are more HR proficient compared to their WT counterparts suggesting that acquired resistant cell lines repair DNA breaks induced by olaparib more efficiently.

Interestingly, some upregulated genes found in RNA processing (*MAGOH*, *SCAF11* and *PLRG*1) have been shown to play a role in tumorigenesis and cancer aggressiveness in glioma, liver, and gastric cancers [28,29,30]. Upregulation of these genes may lead to an aberrant RNA splicing and promote splice-induced gene alterations, such as the Delta11q mutation within the *BRCA1* gene [31]. Some studies in breast cancer showed that targeting RNA splicing with an inhibitor, pladienolide B, reduced the occurrence of BRCA1 mutations that promote acquired resistance in breast cancer [31,32]. A combination of this inhibitor and PARPi may help to limit the development of PARPi resistance in PC.

Autophagy is a complex pathway, which can be pro- or anti-tumorigenic depending on the cancer type [33]. It is now considered a multi-drug resistance (MDR) mechanism in various cancers, including in PC [34,35]. Some studies suggest that autophagy protects PC cells against hormonotherapy and chemotherapy [36,37,38,39]. The impact of autophagy in PARPi resistance has been shown primarily in breast cancer but not in PC [40,41]. We previously reported that the basal level of autophagy can impact the sensitivity to olaparib in PC cell lines [8]. In the present study, upregulation of macroautophagy pathways was observed during our GSEA analyses and expression of the autophagy *ATG2B* gene was significantly increased (Table 3). We did not see any significant variation in RNA or protein expression of ATG2B, but we observed an increase in autophagy basal level in LNCaP-OR and C4-2B-OR cell lines (Figure 4a,b). Some studies suggest that increased levels of autophagy permit cancer cells to repair DNA breaks more efficiently through the HR or BER pathways [41,42,43]. Interestingly, *KIF5B* and *ARL3* found in “protein transport along microtubule” play a role in the last step of autophagy, during the autophagic lysosome reformation (ALR) [44,45]. This possible impact on ALR may lead to an increase in lysosome concentration in cancer cells, which has been shown to induce acquired resistance to the CDK4/6 inhibitor in breast cancer [46]. Based on these analyses, autophagy induction and termination may be important in the olaparib acquired resistance in PC.

Many genes upregulated in the OR cell lines are important in cell cycle regulation (Figure 3d). Among these genes, *ROCK2* plays an essential role in the G1/S phase transition, and studies have shown that its depletion or inhibition leads to both a cell cycle arrest and a senescent phenotype associated with a modification of the composition of the senescence-associated secretory phenotype (SASP) [23,47]. Olaparib is already known to induce a senescence-like phenotype in ovarian cancer cells, which can contribute to tumor progression and acquired PARPi resistance [48,49]. Increased ROCK2 expression may contribute to acquired olaparib resistance due to its impact on senescence. Targeting the protein with a ROCK inhibitor (ROCKi) as a senomorphic drug may limit the induction of the senescence phenotype [50]. Interestingly, the chemical structure of the ROCKi hydroxyfasudil contains the benzamide pharmacophore of PARPi and inhibits the activity of PARP1 and PARP2 by 27% and 50%, respectively [51]. It is already known that senescent cells are resistant to apoptosis by decreasing the expression of pro-apoptotic proteins and increasing the expression of anti-apoptotic proteins [52]. We identified two pro-apoptotic genes, *GRAMD4* and sirtuin-3 (*SIRT3*), with decreased gene expression suggesting a possible negative regulation of apoptosis in the PC OR cell lines.

Our study is limited to the measurement of variations in gene expression between PC WT and PC OR cell lines and gives a first overview of pathways and genes possibly implicated in acquired resistance. Further experiments on ROCK2, either addressing the effects of depletion or overexpression, would need to be conducted to conclude if this protein is important for the resistance seen in PC OR cell lines. Moreover, confirmatory experiments involving the inhibition of autophagy or HR are also necessary to determine their effect on the acquired resistance to olaparib. As in vitro experiments have limitations, it will be important to verify the status of specific pathways and genes directly in tissues from patients resistant to olaparib to understand their clinical relevance.

## 5. Conclusions

To summarize, our study provides a set of PC cell lines with varying degrees of olaparib resistance to study acquired PARPi resistance in PC. Our microarray analysis showed that three major pathways, DNA repair, particularly double-strand DNA breaks, autophagy and senescence, were potentially upregulated in OR cell lines. We also showed by functional assays alterations in DNA repair and autophagy in OR cell lines. Targeting the *ROCK2* gene, pathways such as autophagy or HR may present an opportunity to reverse acquired resistance and reinforce the effect of PARPi in prostate cancer.

## Figures and Tables

**Table 1:** Primers set used for qPCR.

*ß-actin*	Reverse primer Forward primer	5′-CTCCTTAATGTCACGCACGAT-3′ 5′-CATGTACGTTGCTATCCAGGC-3′
*ATG2B*	Reverse primer Forward primer	5′-CTGCATGGGTCGATTTTTCCT-3′ 5′-GGACGGTTAATTGGTAGGTTGG-3′
*BRCC3*	Reverse primer Forward primer	5′-GCTTGTGTGCGAACATCAACA-3′ 5′-GAGTCTGACGCTTTCCTCGTT-3′
*ROCK2*	Reverse primer Forward primer	5′-CCAGGGGCTATTGGCAAAGG-3′ 5′-TCAGAGGTCTACAGATGAAGGC-3′

**Table 2:** Comparison of IC50 values for olaparib between PC wild-type (WT) and olaparib-resistant (OR) cell lines.

	Olaparib (µM)		
Cell Lines	WT	OR	*p* Value
LNCaP	0.12 ± 0.084	0.53 ± 0.11	0.050
C4-2B	0.027 ± 0.018	0.78 ± 0.021	0.024
DU145	7.7 ± 0.48	29 ± 4.27	0.0080

**Table 3:** Top 20 genes upregulated in OR vs. WT PC cell lines.

Name	Gene ID	Fold Change (log2)	Significance (log(−10))
*MYPOP*	339344	0.626066733	3.260994375
*SURF1*	6834	0.971457809	3.133881885
*TAS2R31*	259290	1.594891664	3.03187479
*ROCK2*	9475	1.814688581	3.017431335
*EML5*	161436	0.812352557	2.921011236
*ZNF813*	126017	1.27017308	2.90592896
*PLRG1*	5356	0.922899822	2.890174563
*BHLHB9*	80823	0.881211264	2.819600492
*ANKRD26*	22825	1.316471381	2.743111191
*RANBP1*	19385	1.050488081	2.678150499
*ATG2B*	76559	1.654462509	2.65188022
*SYNJ1*	8867	1.01210112	2.616349744
*RAD54B*	25788	1.309476495	2.543401448
*CAPN14*	440854	0.654425569	2.501443269
*PIK3R4*	30849	0.699793454	2.40793807
*ZNF611*	81856	0.765653399	2.369207217
*CD79B*	974	0.967579395	2.353033565
*KRTAP4-8*	728224	0.716777677	2.350183007
*RPE*	6120	1.061135427	2.324523647
*TRIM59*	286827	1.124953072	2.305366711

**Table 4:** Top 20 genes downregulated in OR vs. WT PC cell lines.

Name	Gene ID	Fold Change (log2)	Significance (log(−10))
*GRAMD4*	23151	−1.455448484	3.820213521
*HIST1H2AM*	8336	−1.079845354	3.038011837
*KRTAP21-1*	337977	−0.678567648	2.665150232
*SERTAD3*	29946	−1.090577359	2.553145699
*DDAH2*	23564	−1.328818688	2.356539512
*LTBR*	4055	−1.102550246	2.295431085
*CCDC92*	80212	−1.533187243	2.290609924
*LOC100133669*	100133669	−0.628676373	2.272973507
*PTK6*	5753	−0.604035851	2.143741754
*ZNF705B*	100132396	−0.705444211	2.081909321
*SLC6A12*	6539	−0.939695006	2.068809797
*ENPP2*	5168	−0.681866615	2.061059338
*LNP1*	348801	−0.899081921	2.05980048
*ACHE*	43	−0.756301787	2.059638325
*SLC35C2*	51006	−1.040655038	2.03265381
*NUDT18*	79873	−1.468949749	2.018578713
*TMEM74*	157753	−0.697450193	2.015048192
*INAFM1*	100688014	−1.31761451	2.011847558
*TRPV4*	63873	−0.632871399	1.991633619
*ARFRP1*	76688	−0.800853082	1.98658451

**Table 5:** Top 10 genes that were enriched in the GSEA gene set for DNA repair.

Name	Gene ID	Change	Fold Change (log2)	Significance (log(−10))
*BRCC3*	79184	Increased	1.17524346372176	1.86861052936291
*WRN*	7486	Increased	1.3897294100393	2.09404094346967
*USP45*	85015	Increased	2.16059849815779	1.51924844116418
*XPA*	395659	Increased	1.31482769069977	1.33272962036067
*XPC*	22591	Unchanged	1.27263828634861	1.15306119992332
*VCPIP1*	428359	Unchanged	0.776414270017991	1.28055627682958
*PRKDC*	5591	Unchanged	2.44036683749613	1.09824709135632
*MBD4*	17193	Unchanged	1.0822056611492	1.25074003757324
*RAD18*	56853	Unchanged	1.12222858709984	1.29614400190726
*PRIMPOL*	201973	Unchanged	1.27599396704602	1.06890196790282

## Data Availability

The datasets used and/or analyzed during the current study are available from the corresponding author on reasonable request. The microarray dataset was deposited to ArrayExpress with accession number E-MTAB-11989.

## Supplementary Materials

The following supporting information can be downloaded at: https://www.mdpi.com/article/10.3390/cancers14163877/s1, Figure S1: Genes affected by OR transformation; Figure S2: Common enriched pathways in all three PC-OR cell lines; Figure S3: Uncropped Western blots.
