# Supplementary material for: Development of Olaparib-Resistance Prostate Cancer Cell Lines to Identify Mechanisms Associated with Acquired Resistance
Source: Cancers (Basel). 2022 Aug 11;14(16):3877. doi: 10.3390/cancers14163877 (PMC9405809; doi:10.3390/cancers14163877)
Supplement: Supplementary file 1 [file cancers-14-03877-s001.zip › cancers-1846898-supplementary.pdf]

Supplementary Materials: Development of Olaparib-resistance Prostate Cancer Cell Lines to Identify Mechanisms Associated with Acquired Resistance

Maxime Cahuzac, Benjamin Péant, Anne-Marie Mes-Masson and Fred Saad

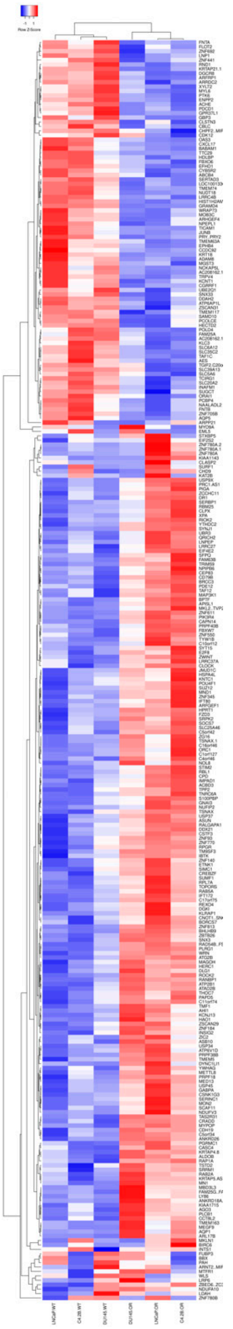

Figure S1. Genes affected by OR transformation. Complete heatmap from Fig. 3c.

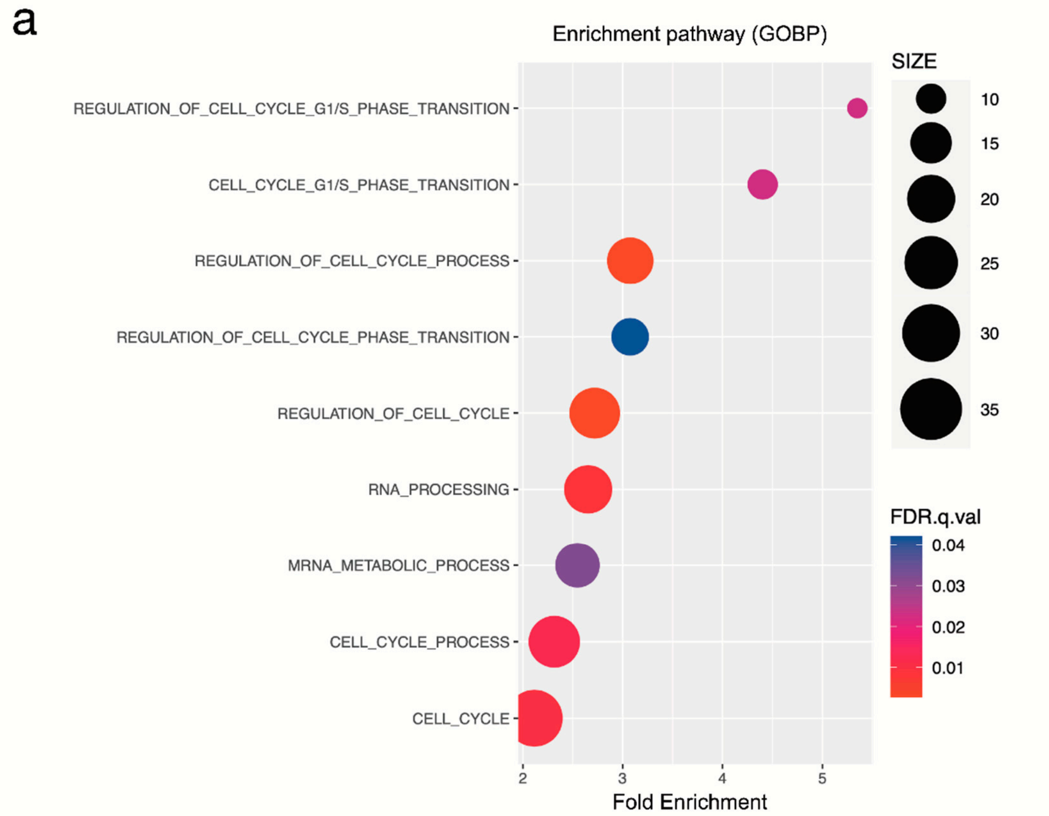

Figure S2. Common enriched pathways in all three PC-OR cell lines. Bubble chart regrouping the most significantly enriched pathways in common in all three PC-OR cell lines determined by GSEA analysis.

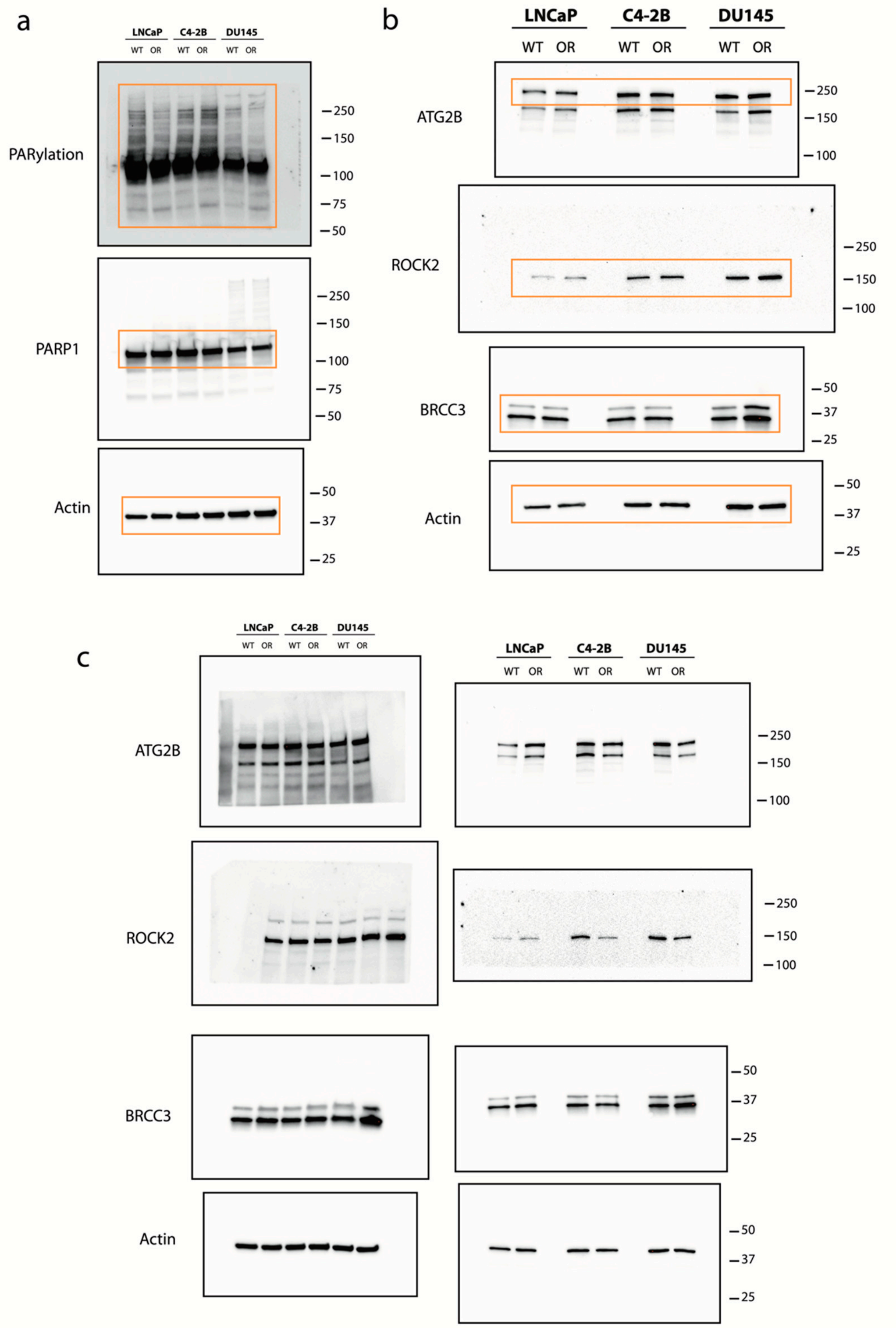

Figure S3. Uncropped Western blots. (a). Western blots from Figure. 1d. (b). Western blots from Figure. 3e. (c). Western blots n = 2 and n = 3 for quantification in Figure. 3f.
